# The Impact of Microbiome on Breast Cancer and Regulatory Strategies

**DOI:** 10.3390/microorganisms14010075

**Published:** 2025-12-29

**Authors:** Jiaxin Wang, Dongyan Xu, Shiyao Hu, Beiwen Zheng, Yiding Chen, Tao Pan

**Affiliations:** 1 Department of Breast Surgery, The Second Affiliated Hospital, Zhejiang University School of Medicine, Hangzhou 310009, China; 22418493@zju.edu.cn (J.W.); 22218720@zju.edu.cn (D.X.); hushiyao@zju.edu.cn (S.H.); 2 State Key Laboratory for Diagnosis and Treatment of Infectious Diseases, Collaborative Innovation Center for Diagnosis and Treatment of Infectious Diseases, The First Affiliated Hospital of Medical School, College of Medicine, Zhejiang University, Hangzhou 310058, China; zhengbw@zju.edu.cn; 3 Jinan Microecological Biomedicine Shandong Laboratory, Jinan 250022, China

**Keywords:** breast cancer, immune, inflammation, microbiome, mechanism

## Abstract

Breast cancer, the most prevalent malignant tumor in women, is closely linked to the human microbiota. The microbiome participates throughout breast cancer pathogenesis, including its occurrence, progression, response to anti-tumor therapies, and treatment-related complications. This review examines the central hypothesis that microbiome-driven inflammatory and immune mechanisms shape breast cancer progression through two key pathways: systemic immune-inflammatory regulation and local tumor microenvironment remodeling. Furthermore, microorganisms and their metabolites modulate systemic treatments by interfering with drug metabolism and altering systemic or local immune-inflammatory environments. Targeting the microbiota represents a promising strategy for enhancing anticancer efficacy and reducing treatment-related complications. This review aims to advance the understanding of the etiology and disease progression of breast cancer from the perspective of microbial-regulated inflammation and immunity, offering new insights for its prevention and treatment.

## 1. Introduction

The human body harbors a vast microbial community, whose gene count exceeds that of the human genome by a hundredfold. The microbiome plays a crucial role in tumor occurrence and progression. Breast cancer (BC) remains one of the most prevalent malignancies globally [1], with its development widely attributed to the interplay of genetic [2,3], environmental, and lifestyle factors [4,5]. Research into the microbiome’s role in breast cancer dates back to 1971, with the pioneering work of M. J. Hill and colleagues [6]. Accumulating evidence supports a correlation between microbial dysbiosis and BC pathogenesis.

The breast and gastrointestinal microbiomes constitute a critical micro-ecosystem regulating breast health. The resident breast microbiome refers to the endogenous microbial community colonizing breast tissue, while the gastrointestinal microbiome encompasses microbial populations from the oral cavity to the colon, with the gut microbiome representing the body’s largest and most functionally active microbial reservoir. The resident breast microbiome is thought to primarily affect local processes such as host cell proliferation and apoptosis, tumor immunity, and drug metabolism [7]. It also exhibits unique functions, including reorganizing the actin cytoskeleton, protecting circulating tumor cells from shear stress, and enhancing cellular motility, thereby promoting metastasis [8]. In contrast, the gut microbiome and bacterial components influence BC systemically [9,10]—by modulating estrogen [11] and lipid [12] metabolism, triggering chronic inflammation, and activating immune responses. Inflammatory mediators, immune cell activation, and gut barrier disruption can further drive tumor progression and affect treatment outcomes.

Among these mechanisms, immune regulation represents a key pathway through which the microbiome affects BC, a link further supported by clinical data on the role of inflammation in BC.

**Figure 1:**
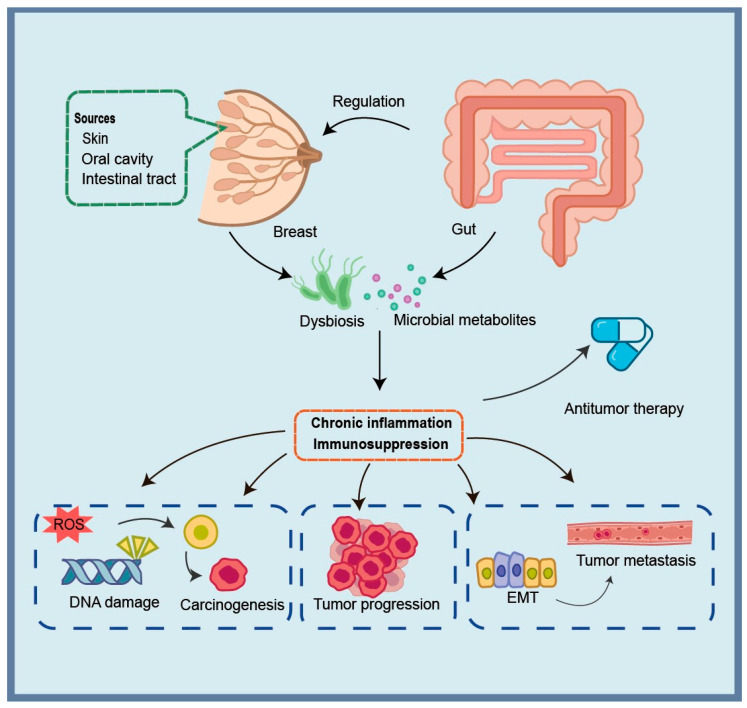
Summary of the impact of microbial-induced inflammation during the development of breast cancer. Microbiome and microbial metabolites from the breast tissue and gut play a dominant role, which may induce chronic inflammatory responses or immune suppression.

This review focuses on how the microbiome participates in BC occurrence, progression, treatment response, and complications via inflammatory and immune pathways. It synthesizes recent findings and related mechanisms (Figure 1), summarizes microbiome-targeted therapies, and explores new perspectives for BC prevention and treatment from a microbiological standpoint.

## 2. Literature Search

To systematically synthesize evidence on how the microbiome influences female breast cancer through immune-inflammatory mechanisms, a comprehensive literature search was conducted across the electronic databases PubMed, Google Scholar, and Web of Science. The search covered publications from 1971 to November 2025. Keyword combinations “immune”, “inflammation”, “breast microbiome”, “gut microbiome”, and “female breast cancer”. After the initial retrieval, titles and abstracts were screened to identify original research and key reviews examining the role of the microbiome, particularly breast and gut microbiota, in breast cancer initiation, progression, and treatment via immune-inflammatory pathways. Papers aligned with the aims of this review were subsequently selected for in-depth analysis and synthesis.

## 3. Microbial Dysbiosis in Breast Cancer Patients

### 3.1. Sources of Breast Microbiome and Dysregulation

#### 3.1.1. Possible Origin of the Breast Microbiome

Breast tissue has been shown to host unique and diverse microbial communities, including bacteria and viruses, collectively known as the breast microbiome [13]. By utilizing next-generation sequencing technology, studies have demonstrated that the microbial composition of breast tissue in women without breast infections is similar to, but distinct from, that of the skin and gastrointestinal tract [14,15]. Regarding the origin of the breast microbiome, current hypotheses indicate two potential sources: microbial communities from the skin or oral cavity may directly enter through the open nipple, or alternatively, migrate from the gastrointestinal tract [16,17]. It has been hypothesized that intestinal bacteria can escape through intercellular gaps of epithelial cells and migrate to the breast via the bloodstream or lymphatic system. Alternatively, they can possibly be carried by dendritic cells (DCs) and migrate to distant sites without disrupting the tight junctions of the mucosa [16]. These findings not only shed light on the potential origins of the breast microbiome but also provide critical insights for further investigating its role in the initiation and progression of BC.

#### 3.1.2. The Specific Types and Locations of the Microbiome in Breast Tumor Tissues

Analysis of the microbial composition of breast skin and breast tissue has revealed that the predominant microbiota in the breast are Pseudomonadota, Bacillota, and Actinomycetota [14,18]. However, the microbial composition differs significantly between healthy and cancerous tissue. Using 16S rRNA sequencing technology, Heiken et al. demonstrated a significant decrease in the microbial alpha-diversity (α-diversity) in BC tissue [19]. α-diversity is a measure of within-sample microbial diversity capturing both taxonomic richness and evenness. The microbiome of healthy individuals’ breast skin swabs and breast tissue is enriched with *Prevotella*, *Streptococcus*, *Micrococcus*, and *Lactobacillus* [18]. Alice Tzeng et al. discovered that *Propionibacterium* and *Staphylococcus* are significant components of healthy control and tumor-adjacent normal types but are rarely found in tumor tissues [20]. In contrast, Camilla Urbaniak reported higher concentrations of *Staphylococcus* in tumor tissues [18]. Although *Staphylococcus* represents one of the most abundant and prevalent genera in breast tissue, its distribution and role in BC remain controversial, necessitating further research to elucidate its relationship with the tumor microenvironment (TME) [21].

BC patients, conversely, exhibit an increased abundance of specific microbiota, including *Clostridium*, *Bacillus*, Enterobacteriaceae, and *Pseudomonas*. Microbial alterations within the mammary duct are further supported by nipple aspirate analysis, which reveals compositional shifts during malignant progression. For instance, 16S rRNA sequencing of nipple aspirate fluid detected *Alistipes* exclusively in breast cancer patients, while noting a significant reduction in an unclassified genus from the Sphingomonadaceae family [22]. Additionally, microbial composition varies significantly across molecular subtypes [23] (Table 1).

Note: Definitions of tissue types in this table:

- Healthy Control Tissue: Breast tissue obtained from individuals without breast cancer (e.g., from reduction mammoplasty), serving as the control baseline.
- Tumor Tissue: Pathologically confirmed breast cancer lesion tissue.
- Tumor Adjacent Normal Tissue: Tissue obtained from breast cancer patients, located outside the visible tumor margin (typically ≥ 5 cm) and pathologically confirmed to be free of cancer cell infiltration, considered “normal” breast tissue from a cancer-bearing host.

Early studies have confirmed the existence of live bacteria within solid tumors through bacterial culture and the detection of bacterial-specific components, such as D-alanine, a key constituent of bacterial cell walls [8,23]. Deborah Nejman’s team, using optimized qPCR and 16S rRNA sequencing technologies, detected extremely high bacterial loads in the breast tissue of mice with BC. This not only confirmed the dysbiosis of specific microbial populations in the breast but also demonstrated that bacteria were primarily localized in the perinuclear cytoplasm of tumor cells and immune cells through FISH targeting 16S rRNA and immunohistochemical (IHC) targeting lipopolysaccharide (LPS) and lipoteichoic acid (LTA) [23]. Correlative light and electron microscopy (CLEM) further confirmed these distribution patterns. These studies suggest that bacteria in the TME are predominantly present as “intracellular bacteria”.

Epidemiological evidence has identified potential associations between BC and five viruses: human mammary tumor virus (HMTV), human papillomavirus (HPV), Epstein–Barr virus (EBV), bovine leukemia virus, and human cytomegalovirus (HCMV) [27,28]. However, significant controversy remains regarding whether viruses or other pathogens play a role in human breast cancer. A key point of debate is the extremely low abundance of viral DNA detected in breast tumor tissues. Thus, conclusive evidence supporting a direct role of these viruses in human breast carcinogenesis remains lacking [29].

Current research on fungi in relation to BC remains relatively limited, but recent studies have begun to uncover its potential mechanisms. A typical example is that breast cancer patients with higher abundance of *Malassezia globosa* (*M. globosa*) in tumor tissues exhibit significantly shorter overall survival (OS). A large-scale pan-cancer analysis of fungal ecology in breast tissue and blood revealed not only the presence of fungal communities but also their physical and biochemical interactions with bacteria, proposing the concept of “fungal–bacterial–immune clusters” within the TME [30]. In BC samples, inter-domain co-occurrence associations were particularly prominent, with *Aspergillus* and *Malassezia* acting as key hubs in the co-occurrence network. Imaging analysis further confirmed that most fungi were located within cancer cells, resembling the localization pattern of intracellular bacteria [30].

### 3.2. Dysbiosis of Gut Microbiota and Its Mechanisms in Breast Cancer

#### 3.2.1. Digestive Tract Microbiota Dysbiosis

Gastrointestinal microbiotas collectively shape a complex immune regulatory system. The gut microbiota plays a critical role in maintaining immune homeostasis and internal equilibrium within the body. However, prolonged dysbiosis of this microbial community may contribute to the pathogenesis of diseases by compromising intestinal barrier function and inducing chronic immunosuppressive inflammation [31]. Breast cancer patients exhibit distinct gut microbiota alterations characterized by reduced α-diversity and specific microbial shifts [32]. Compared to healthy controls, BC patients show increased abundance of Fusobacteriota, such as *Fusobacterium nucleatum* (*F. nucleatum*), alongside decreased Bacillota, such as *Ruminococcus*, *Faecalibacterium prausnitzii* (*F. prausnitzii*), and Bacteroidota (*Odoribacter*, *Butyricimonas*) [32,33]. Postmenopausal patients demonstrate unique patterns with enrichment of potentially pathogenic species (*Escherichia coli* (*E. coli*), *F. nucleatum*) and depletion of protective bacteria (*Eubacterium eligens*, *Lactobacillus vaginalis*) [34]. These findings suggest gut microbial dysbiosis may play a role in BC development and progression.

#### 3.2.2. Unique Status of the Gut Microbiome

The gut microbiota plays a critical role in maintaining the body’s immune homeostasis and internal environment balance. Dysbiosis of the gut microbiota can lead to the production of inflammatory mediators and activation of inflammatory cells, accompanied by damage to the intestinal mucosal barrier. The intestinal mucosa possesses a unique immune system, primarily composed of mucosa-associated lymphoid tissue (MALT) and various immune cells. DCs in the intestinal lamina propria recognize antigens and present them to T cells generated in Peyer’s patches, resulting in inflammation and inducing T cell differentiation into immunosuppressive regulatory T cells (Tregs).

Additionally, bacterial components or metabolites, such as short-chain fatty acids (SCFAs), lithocholic acid, and folate, can exert local effects or enter the systemic circulation to exert distal effects [9,10]. In these ways, dysbiosis of the gut microbiome can induce inflammatory responses and immune suppression.

### 3.3. Oral Microbiome and Breast Cancer Risk

Although studies specifically investigating the oral microbiota–breast cancer relationship remain limited, emerging evidence suggests that poor oral hygiene and related pathologies are associated with an elevated risk of breast cancer. For instance, tooth loss has been identified as a potential risk factor for breast carcinogenesis [35]. Periodontitis, primarily mediated by Gram-negative bacteria such as *Porphyromonas gingivalis* (*P. gingivalis*), *F. nucleatum* and *Prevotella intermedia,* is believed to promote oncogenesis through systemic inflammation and recurrent bacteremia [36]. This bacteremia facilitates the translocation of oral microbes into the circulation, enabling species such as *F. nucleatum*—a Gram-negative anaerobe originating from the oral and gastrointestinal tracts—to potentially colonize breast tissue via hematogenous dissemination [37].

Furthermore, microbial profiling has revealed taxonomic shifts in the oral microbiota of breast cancer patients. Among East Asian cohorts, specific oral bacterial genera, such as *Pasteurellaceae*, *Streptococcaceae*, and *Fusobacteriaceae*, show relatively higher abundance, whereas *Bacteroidaceae* shows lower abundance compared to other taxa [38]. However, various studies have shown that the correlation between oral microbiome and breast cancer is still uncertain and contradictory [38,39]. Given the taxonomic and functional overlaps between the oral and gut microbiomes, as well as shared pathophysiological mechanisms, investigating the oral microbiota offers a valuable complementary perspective to understanding the gut microbiome’s role in BC biology.

### 3.4. Current Landscape and Methodological Challenges in Breast Microbiome Research

The human microbiome, encompassing both breast tissue and the gastrointestinal system, constitutes a dynamic and complex ecosystem [19,40,41,42]. Its composition and diversity are continuously shaped and regulated by multidimensional factors throughout an individual’s lifespan: exogenous factors (such as dietary patterns, geographical environment, and medication use) exhibit a certain degree of modifiability, whereas endogenous factors (such as host genetic background and immune status) form a relatively stable biological foundation. However, due to significant heterogeneity among studies in terms of population characteristics, sequencing techniques, and bioinformatic analyses, the results at the genus level vary considerably across different investigations, posing challenges for conducting reliable meta-analyses [21,42,43].

Furthermore, as breast tissue is a low-biomass environment, microbiome research on it is highly susceptible to contamination, and the commonly used 16S rRNA sequencing technique has its limitations [44]. Therefore, stringent negative controls and multi-omics validation are crucial for drawing reliable conclusions.

Despite the existing heterogeneity across studies, the association between certain specific microorganisms and breast cancer has gained broad empirical support, with the research focus gradually expanding from disease association mechanisms to microbiota-mediated therapeutic strategies.

## 4. The Microbiome and the Occurrence, Development, and Metastasis of Breast Cancer

**Figure 2:**
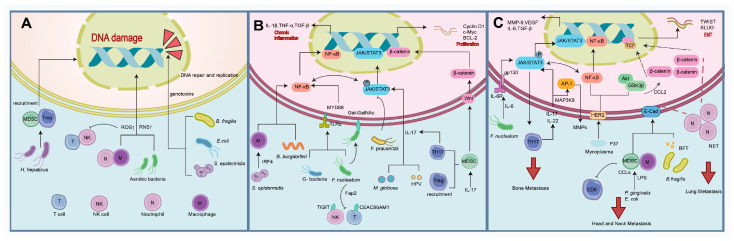
Schematic overview of the multifaceted mechanisms contributing to (**A**) DNA damage and carcinogenesis, (**B**) tumor progression, and (**C**) distant metastasis.

The specific breast microbiome can modulate the TME by promoting local inflammation or immune responses, thereby inducing DNA mutations, activating oncogenic pathways, and initiating distant metastasis. In parallel, the gut microbiome contributes to TME reprogramming and signaling pathway dysregulation primarily by triggering systemic inflammation and generating bioactive metabolites. The key mechanisms discussed in this article are summarized in Figure 2.

### 4.1. Initiating Receptors of Microbiome-Related Inflammatory Responses

The inflammatory response triggered by the microbiome generally begins when pathogen-associated molecular patterns (PAMPs) are recognized by pattern recognition receptors (PRRs) on antigen-presenting cells (Table 2).

Toll-like receptors (TLRs) are the most important among PRRs [45]; others include NOD-like receptors (NLRs) and RIG-I-like receptors (RLRs). The TLR family consists of 13 members, each with distinct patterns. For instance, TLR1, TLR2, and TLR6 can recognize lipoproteins from Gram-positive bacteria, TLR4 recognizes LPS, and TLR5 is the only member of the TLR family that recognizes flagellin [46]. Pathologists have systematically studied the expression of TLRs in breast cancer cell lines and tissues, finding differences in the levels and types of TLR expression in different cells. For example, the expression of TLR2 is higher in MDA-MB-231 cells than in MCF-7 cells, while the expression of TLR3 is lowest in the MDA-MB-231 cell line [47]. Studies indicate that TLRs may be associated with the development and prognosis of breast cancer. The elevated expression of TLR3, TLR4, and TLR9 is associated with poorer survival in breast cancer patients [48]. NOD1 binds to microbial components such as bacterial peptidoglycan and activates innate immune responses via the NF-κB or MAPK signaling pathways [49]. RLRs are cytosolic RNA sensors crucial for detecting viral RNA, but their role in breast cancer is not well studied [50].

Formyl peptide receptor-1 (FPR1) is another PRR that is typically highly expressed in DCs. A single nucleotide polymorphism, Rs867228, can cause its loss of function, increasing susceptibility to infections caused by bacteria such as *Listeria monocytogenes*, *Staphylococcus aureus*, *Streptococcus pneumoniae* and *E. coli*, thereby increasing the risk of luminal B-type breast cancer [51].

### 4.2. Microbiome-Induced Genomic Instability and Tumor Initiation: Roles of Inflammation and Immunosuppression

#### 4.2.1. Direct Genotoxic Bacterial Toxins

The induction of DNA mutations is one of the key mechanisms by which the microbiome initiates tumorigenesis. Studies isolating bacteria from breast tissue have found that pks-positive *E. coli* (secretes colibactin [52]) and *Staphylococcus epidermidis* (*S. epidermidis*) can induce DNA double-strand breaks in breast epithelial cells by producing genotoxins. This type of DNA damage is one of the most severe forms, thereby causing genomic instability [18]. Genomic instability is a hallmark of cancer initiation, as it accelerates the accumulation of mutations in oncogenes and tumor suppressor genes, thereby driving malignant transformation. Additionally, enterotoxigenic *Bacteroides fragilis* (ETBF) secretes the *B. fragilis* toxin (BFT), a virulence factor that epigenetically reprograms mammary epithelial cells and drives mutagenesis, targeting key tumor suppressor genes such as NF2, FAT4, RSK3, DCN, and DOK2. The downregulation of these genes has been linked to poor prognosis in human breast cancer [53]. In mouse models, BFT exposure promotes the development of multifocal breast tumors with elevated stem-like properties and induces prominent morphologic and functional alterations in both normal and malignant breast epithelial cells [54].

#### 4.2.2. Immune-Driven Oxidative DNA Damage

Microbiome-induced immunosuppression represents another mechanism driving genomic instability in breast cells. This occurs primarily through the induction of elevated levels of reactive oxygen species and reactive nitrogen species (ROS/RNS), which are potent mediators of DNA damage [55]. Extensive research has shown that microbial infections such as *F. nucleatum*, *Helicobacter*, and *Enterococcus faecalis* are closely associated with elevated levels of ROS and DNA damage, which play significant roles in human carcinogenesis [56,57]. In this setting, beyond intrinsic aerobic metabolism [58], the dominant source of ROS/RNS is the host immune response. During microbial infection, recruited and activated immune cells, such as neutrophils and macrophages, produce substantial quantities of superoxide, diverse radicals, and nitric oxide (NO). These reactive species can oxidize nucleobases, attack the DNA backbone, and interfere with DNA replication and repair processes [59]. Furthermore, specific bacteria can directly induce ROS production in host cells. The ETBF colonizes both the mammary gland and the gut, where it activates the NF-κB signaling pathway and induces ROS production [60].

Apart from the direct impact on BC cells and DNA mutations, ROS and RNS can in turn drive TME [61], shape a pro-cancer microenvironment, activate important signaling pathways such as NF-κB and MAPK, inhibit the function of natural killer cells (NK cells) and T cells, and recruit immunosuppressive cells such as Tregs or myeloid-derived suppressor cells (MDSCs). This recruitment leads to increased secretion of inflammatory factors [62,63], ultimately progressing to full malignancy [64]. In the C3(1)-Tag breast cancer mouse model, *Helicobacter hepaticus* (*H. hepaticus*) can translocate from the gut to the breast and promote the recruitment of MDSCs and neutrophils [65]. This process is mediated by the activation of signaling pathways such as activator protein 1 (AP-1), Wnt/β-catenin, and the upregulation of cyclin D1, which collectively induce a pro-tumor inflammatory microenvironment infiltrated by TNF-α and Tregs, thereby promoting mammary tumorigenesis [66].

### 4.3. Microbiome-Driven Mechanisms of Breast Cancer Progression

The microbiome can drive BC initiation and progression by modulating host signaling pathways and immune responses. Key mechanisms include sustained activation of critical inflammatory pathways such as STAT3 and NF-κB, coupled with recruitment of immunosuppressive populations like M2 macrophages and MDSCs, collectively fostering a pro-tumorigenic and immune-evasive microenvironment.

Under pathogenic conditions, STAT3 overexpression drives breast cancer cell progression and proliferation while inhibiting apoptosis and orchestrating immune suppression [67]. Moreover, in HPV-positive BC patients, tumor tissue exhibits higher levels of IL-6, IL-17, TNF-α, and TGF-β, along with activation of NF-κB and STAT3 [68,69]. This indicates that HPV may serve as a potential risk factor contributing to tumor progression. *F. prausnitzii* colonizing the gut can inhibit the growth of MCF-7 cells by inhibiting IL-6 secretion and JAK2/STAT3 phosphorylation [70].

NF-κB and STAT3 are frequently co-activated in the TME and collaboratively mediate the microbiome-driven promotion of tumorigenesis [71]. Under conditions of microbial dysbiosis, the upregulation of TLR signaling in breast cancer cells activates the NF-κB pathway, leading to the secretion of inflammatory cytokines, which may stimulate the proliferation of breast tumor cells. For example, *Borrelia burgdorferi* (*B. burgdorferi*) infection of MDA-MB-231 cells can lead to upregulation of the chemokine family (CXCL1, 2, 3, 8, and 10) and CCL20 and activate NF-κB signaling, increasing the secretion of inflammatory factors IL-3, 4, 10, and 17 [72], collectively contributing to cancer progression. A recent study found that *M. globosa* colonization in breast tissue promotes the production of IL-17A by breast cancer cells, modulates NF-κB activity, and increases the expression of IL-6 and cyclooxygenase-2 (COX-2), thereby stimulating the proliferation of mice BC cells [73,74]. Studies have shown that *F. nucleatum* can recognize human cell Gal-GalNAc via adhesin (Fap2) [75], thereby colonizing breast tumor tissue and inducing chronic inflammatory responses. It also activates the TLR4/MyD88 pathway in breast cancer, leading to the upregulation of NF-κB pathway-associated cytokines such as IL-1β or TNF-α, exerting direct or indirect immunosuppressive effects [76,77]. Therefore, in cancer samples containing this bacterium, both the content of T cells and the activity of NK cells have decreased [75]. The possible reasons include the binding of Fap2 to immune inhibitory receptors, TIGIT [78] and CEACAM1 [79], which leads to the inhibition of immune cell function.

Furthermore, the microbiome promotes tumor progression by remodeling the immune cell composition. M2 macrophages represent a major immunosuppressive population in the mammary microenvironment, facilitating immune escape through recruitment of Tregs and suppression of effector T cell function [80]. Through the analysis of 16S rRNA gene sequencing, *S. epidermidis* has been isolated from mammary tumors in 4T1 tumor-bearing mice. Despite its typical association with cellular DNA damage, it can also induce inflammation, upregulate interferon regulatory factor 4 (IRF 4), promote M2 macrophage polarization, activate the complement pathway, and cause the secretion of pro-inflammatory cytokines IL-1β, IL-6, IL-18, TNF-α, and chemokines MCP-1, MIP-1α, MIP-2, RANTES, EOTAXIN, and GROα, ultimately inducing an inflammatory TME and tumor immune suppression [51,81]. Elevated serum IL-10 levels have been observed in BC patients. HCMV expresses a viral homolog of IL-10, which is considered a potential mechanism through which this virus may promote BC development [28,82]. Meanwhile, HCMV infection in breast tissue alters the phenotype and transcriptional profile of macrophages, driving their polarization toward an M2 phenotype [83]. Similarly, gut dysbiosis orchestrates the infiltration of myeloid cells (notably macrophages) and a pro-inflammatory state within mammary tissue, which collectively advances BC progression [84].

Microbial and inflammatory dysregulation recruits MDSCs to induce an immunosuppressive environment, thereby promoting cancer progression [85,86]. MDSCs enhance immune evasion through multiple mechanisms: they expand Tregs and Th17 cells [87], while IL-17 and IL-6 [88] reciprocally promote MDSC infiltration into tumors [89], establishing a feed-forward loop that sustains a pro-tumorigenic niche.

### 4.4. Core Mechanisms Linking Inflammation to Tumor Metastasis

The chronic inflammatory microenvironment, shaped by both local breast and gut microbiota, is a critical driver of breast cancer metastasis. This microenvironment facilitates metastasis through multiple mechanisms, including the induction of epithelial–mesenchymal transition (EMT) in tumor cells and systemic immune modulation [84,90].

Recent studies have identified a correlation between increased abundance of *Staphylococcus* and *Lactobacillus* within breast tumors and elevated lung metastasis burden in mouse models. Experimental findings indicate that antibiotic treatment reduces intratumoral microbial load and decreases lung metastases without affecting the growth of primary tumors [81]. Additionally, *Helicobacter hepaticus* DNA has been detected in peritumoral lymph nodes of infected mice, suggesting microbial dissemination via lymphatic routes [20]. Studies also found that lymph node positivity was positively correlated with *Acinetobacter* and *Bacteroides* but negatively correlated with *Achromobacter*, whereas lymphovascular invasion was positively correlated with *Lactobacillus* and negatively correlated with *Alkanindiges* [20]. Biopsy analysis of human BC metastatic sites revealed that PD-L1 expression and tumor-infiltrating lymphocyte (TIL) levels are negatively correlated with microbiome diversity. Tumors with lower microbiome diversity typically show higher PD-L1 expression and more TIL infiltration; however, these patients have a poorer prognosis. This supports the “Cancer–Microbiome–Immune Axis” hypothesis [91].

The overlap in signaling pathways between the breast microbiome-induced chronic inflammation and the TME suggests that this inflammatory milieu may play a role in both initiating and sustaining EMT in tumor cells [92]. For instance, factors such as IL-1β, IL-6, and TNF-α can affect the expression of EMT-related transcription factors like Twist and Slug, and the synergistic expression of TNF-α, IL-1β, and CCL2/CCL5 can enhance EMT [93]. *F. nucleatum* can induce EMT and tumor cell stemness via the IL-6/STAT3 signaling pathway [94]. BTF can also upregulate EMT-related Slug and Twist, induce epithelial hyperplasia, promote E-cadherin cleavage and activate the β-catenin and Notch1 pathways to enhance cell migration [54,95]. Mycoplasmal lipoprotein p37 binds to mammalian proteins EGFR and HER2, activating downstream Erk1/2, Akt, and NF-κB pathways, thereby conferring anti-apoptotic properties and promoting metastasis [96].

IL-17-mediated pro-inflammatory TME promotes tumor metastasis. Specific gut or intratumoral microbiota (e.g., segmented filamentous bacteria) may modulate BC progression via the IL-17 pathway by promoting Th17 differentiation and increasing IL-17 secretion in mouse models [97]. Additionally, a study has shown that IL-22 released by Th17 cells can activate the MAP3K8 signaling pathway in BC cells, which in turn activates the STAT3 and AP-1 pathways, thereby increasing breast cancer aggressiveness [98]. IL-17 plays a crucial role in the immune microenvironment. Specifically, the IL-17A/NF-κB/MMPs axis promotes breast cancer bone metastasis [99].

The microbiota can create a favorable environment for distal metastasis by recruiting immune cells and activating inflammatory networks. Following intestinal microbiota dysbiosis, host cells release chemokines (CC and CXC ligands), microbial metabolites, and recruitment of MDSCs promotes tumor EMT and metastasis. Both mouse periodontitis models and human gingival fibroblasts stimulated by *P. gingivalis* or *E. coli* LPS exhibit increased production of IL-1β in the gingiva. This inflammatory response further promotes the secretion of chemokines such as CCL2, CXCL5, and CCL12, thereby recruiting MDSCs and macrophages [36], fostering both local and systemic inflammatory microenvironments associated with head and neck metastasis. A recent animal study found that respiratory infections caused by viruses such as influenza and SARS-CoV-2 can trigger the activation of dormant breast cancer cells in the lungs via the inflammatory cytokine IL-6, suppress T cell activation to induce immunosuppression, and ultimately lead to pulmonary metastasis. These findings have been corroborated by human epidemiological research [100]. In vitro and mouse studies have shown that *M. globosa* promotes tumor growth by activating the pro-inflammatory MBL-C3a-C3aR signaling cascade, which drives macrophage polarization toward an M2 phenotype, thereby enhancing the proliferation, migration, and invasiveness of BC cells [101].

Neutrophils are key effectors in microbiota-mediated metastasis [102]. In mice exposed to antibiotic-induced disturbance of the gut microbiome, severe systemic inflammation and significant neutrophil infiltration were observed in bone metastatic sites, accompanied by tumor cell extravasation and transendothelial migration [103]. Studies have also found that intravenous *Streptococcus* can promote thrombus formation and BC lung metastasis by inducing local neutrophil extracellular trap (NET) formation and vascular inflammation [104]. Furthermore, this process recruits MHCII(hi) neutrophils into the lungs via CCL2, thereby promoting pulmonary metastasis of breast cancer [105].

### 4.5. The Influence of Microbial Metabolites on the Onset and Progression of Breast Cancer

Intestinal bacterial metabolites, such as bile acids, SCFAs and lactate, predominantly exert anti-tumor effects by promoting apoptosis and enhancing immune responses [106], reaching distal tumor sites via the bloodstream (Table 3). LCA (lithocholic acid) [107], indole derivatives [108], and SCFAs [109] can suppress EMT and the metastasis of tumors. In addition, other bacterial metabolites such as acetic acid, butyric acid, ethanol, D-mannitol, 2,3-butanediol, and trans-ferulic acid have also been shown to exhibit inhibitory effects on breast cancer cells [109,110]. In contrast, there are fewer reports on the impact of local breast microbiota and their metabolites on tumor immunity. However, existing studies suggest that specific bacterial metabolites like LPS and microbiota-derived extracellular vesicles (EVs) may promote tumor progression (Table 3).

Lithocholic acid, a major bacterial metabolite, is significantly generated by aerobic flora in the gut, especially clostridia [121]. In patients with early-stage BC, serum LCA levels are often decreased. LCA may primarily inhibit EMT and enhance tumor immunity by acting through the TGR5 receptor on BC cells [107].

Short-chain fatty acids are primarily produced through the metabolic breakdown of carbohydrates by the gut microbiota, with a small portion also derived from amino acid metabolism [122]. SCFAs mainly include acetate, propionate and butyrate. In breast cancer mouse models, Blautia and its metabolite acetate increase tumor-infiltrating CD8+ T cell populations, thereby enhancing anti-tumor immunity and reducing metastasis [111]. Sodium propionate can exert anti-BC effects by inhibiting the JAK2/STAT3 signaling pathway, causing cell-cycle arrest at the G0/G1 phase, increasing ROS levels, and promoting phosphorylation of p38 MAPK, which ultimately induces apoptosis [112]. Butyrate can bind to SCFA receptors like G-protein-coupled receptor 109A (GPR109A) and GPR43 on macrophages and DCs, thereby regulating CD4+ T cell differentiation and activating CD8+ cells [114]. Through these mechanisms, butyrate influences immune cell differentiation and function, lowers chronic inflammation, and restricts tumor growth [123]. Its role is analogous to that of *Prevotella* in maintaining health in normal tissues. Butyrate can also enhance the effectiveness of anti-PD-1 immunotherapy by regulating the signaling pathways in CD8+ T cells [113].

Although lactate is not strictly classified as an SCFA, it is a product of carbohydrate breakdown by *lactobacilli* and can be further metabolized to generate other SCFAs [124]. Lactate has the potential to skew macrophages towards the M1 type and enhance the functionality of CD8+ T cells. When the gut microbiota is dysregulated, a decrease in lactobacilli abundance may create an immunosuppressive TME and increase the risk of breast cancer in mice. However, excessively high concentrations of lactate may reduce the tumor immunity in the TME [37], potentially suppressing anti-tumor activity of immune cells, especially CD8+ T cells [37], and promoting M2 macrophage polarization [115].

Folate is one of the gut microbial metabolites, and the relationship between folate and BC risk remains incompletely understood, with conflicting findings across studies. Early meta-analyses suggested that folate intake has little to no significant effect on overall breast cancer risk [125,126]. However, some studies indicate that folate may be associated with ER subtypes. It can reduce the risk of ER-negative (particularly ER-/PR-) breast cancer without affecting overall breast cancer incidence [127]. Notably, since folate is closely linked to carcinogenesis, its effects may be mediated through epigenetic regulation as well as DNA repair and synthesis [116]. Furthermore, in fecal samples from BC patients with bone metastasis, reduced abundances of *Megamonas* and *Akkermansia* were observed. PICRUSt predicted that the gut microbiota influences host lipid metabolism and folate synthesis, which in turn contributes to breast cancer and bone metastasis [128].

Under normal physiological conditions, estrogens are predominantly eliminated in conjugated forms via urinary and fecal excretion. Emerging evidence indicates that specific intestinal microbiota participate in estrogen metabolism through hydrolytic, reductive, and oxidative reactions, thereby reactivating estrogenic compounds and modulating systemic estrogen bioavailability. Notably, numerous bacterial species express β-glucuronidase and β-glucosidase enzymes, which catalyze the deconjugation of estrogens and facilitate their enterohepatic recirculation and reabsorption into circulation.

Studies have demonstrated that microbiota-mediated estrogen metabolism exerts bidirectional effects on immune function. Transient elevation of estrogen levels enhances NK cell activity, whereas sustained hyperestrogenemia suppresses peripheral NK cell cytotoxicity [129], upregulates Treg frequency, and polarizes macrophages toward an M2-like immunosuppressive phenotype, collectively increasing host susceptibility to tumorigenesis and metastatic dissemination [130,131]. Within the TME, elevated estrogen levels correlate with functional alterations in dendritic cells, macrophages, B cells, and T cells, and stimulate monocytic secretion of pro-inflammatory cytokines, including TNF-α, IL-6, and CXCL8, thereby sustaining a chronic inflammatory milieu conducive to tumor progression [132]. Furthermore, significant gaps remain in understanding the extent of the association and underlying mechanisms between estrogen levels and the immune microenvironment, particularly in different breast cancer subtypes (e.g., ER+/ER-).

Additionally, it is known that metabolites from gut bacteria can enter the bloodstream and reach target organs (breast tissue) to exert biological functions. However, there is currently little conclusive evidence that the mammary ductal microbiota can directly produce such metabolites to affect tumor progression. Only animal studies have suggested that they may promote tumor proliferation through non-immune-related pathways, highlighting a potential difference in the mechanisms of action between gut and breast microbiomes [133,134]. Recent research has found that trimethylamine N-oxide (TMAO) metabolized by *Clostridiales* in breast tissue induces pyroptosis in tumor cells via activating endoplasmic reticulum stress kinase PERK, enhancing CD8+ T cell-mediated anti-tumor immunity in triple-negative breast cancer (TNBC) in vivo [135]. Whether the mammary microbiota employs immune-modulatory mechanisms similar to those of the gut microbiota remains an open question that requires further investigation.

It is noteworthy that LPS, a major component of the outer membrane of Gram-negative bacterial cell walls, can induce EMT by downregulating the Akt/GSK3β/β-catenin pathway via TLR2 and TLR4, thereby increasing the invasiveness of breast cancer [118,119]. The Cyclin D1 pathway may be an important endogenous signaling mechanism in this process [136]. Additionally, research has shown that the LPS/S100A7/TLR4 signaling pathway may promote tumor growth and metastasis by recruiting MDSCs and weakening TLR4-mediated tumor immunity [117].

Within the TME, microbiota-derived EVs play critical roles in intercellular communication. EVs contain a diverse array of biomolecules, including DNA, RNA, lipids and metabolites. These components influence cell–cell communication and contribute to the modulation of inflammation and immune responses [137]. EVs from pathogens carry PAMPs capable of directly activating host immune responses, leading to the release of inflammatory and chemotactic factors, thereby triggering chronic inflammation or immune suppression. Recent studies have shown that small EVs derived from *F. nucleatum* can also promote proliferation and migration of breast cancer cells by activating TLR4 [76]. Conversely, EVs from probiotic bacteria exhibit anti-inflammatory properties. In the future, EVs can serve as early diagnostic biomarkers for breast cancer, including TNBC, by analyzing specific components within them, or be explored for delivering therapeutic agents or genes, aiming for precise therapeutic interventions [120].

## 5. Therapeutic Applications of Microbiome

Currently, the treatment methods for breast cancer include surgery, chemotherapy, radiotherapy, targeted therapy, and endocrine therapy. The microbiome is increasingly recognized to influence the therapeutic effect, primarily by driving drug resistance, shaping immune/inflammatory responses, and reducing adverse effects, as outlined in Table 4. Consequently, strategies to modulate the microbiota or harness its metabolites hold significant translational promise for inhibiting tumor progression, enhancing chemotherapy, reducing side effects, and serving as novel adjuvant strategies.

### 5.1. Impacts of Microbiome on Breast Cancer Therapy

The gut and intratumoral microbiota appear to shape the efficacy of breast cancer chemotherapy and anti-tumor immunity, primarily through putative effects on drug metabolism, immune/inflammatory modulation, and TME remodeling. Regarding the breast microbiome, studies using mouse models have shown that *Mycoplasma* infection within the TME can rapidly decompose gemcitabine via a bacterial cytosine deaminase, thereby reducing its anti-tumor efficacy [138]. More broadly, gut bacteria exert profound systemic regulatory effects on chemical therapeutics through their potent metabolic and immunomodulatory capabilities.

Following cyclophosphamide (CTX)-induced disruption of the intestinal epithelial barrier, specific bacteria (e.g., *Lactobacillus johnsonii*, *Lactobacillus murinus*, *Enterococcus hirae*, and Clostridiales) [139,140,146] migrate through the circulatory system to secondary lymphoid organs [147]. There, they potentiate the anti-tumor immune response by inducing Th17 and Th1 cells, thus enhancing the chemotherapeutic effect of CTX. Germ-free or antibiotic-treated mice show a decrease in Th17 responses and resistance to CTX. One of the mechanisms of action of platinum-based chemotherapy drugs is to induce DNA damage through the generation of ROS and promote apoptosis [148]. This mechanism is intricately linked to the microbial immune regulation within the TME. Previous studies have shown that microorganisms can enhance the efficacy of platinum drugs by modulating the TME. For example, the ROS responsible for the genotoxicity of oxaliplatin (OXA) in vivo mainly comes from tumor-associated inflammatory cells. However, in antibiotic-treated or germ-free mouse models, the expression of pro-inflammatory genes and cytokines is significantly reduced, affecting their therapeutic effect on tumors. Therefore, a balanced intestinal microbiota is essential for the therapeutic effect of OXA via regulating the TME, suggesting that the optimal therapeutic response to cancer treatment depends on a complete commensal microbiota [141]. Similar results were observed in the treatment of anthracyclines, alkylating agents, and other drugs that induce ROS as part of their anticancer mechanism. Antibiotic use is not invariably detrimental; for example, some regimens can modulate the intestinal microbiota to enhance docetaxel efficacy, a process wherein an increase in *Akkermansia muciniphila* (*A. muciniphila*) abundance plays a significant role [142].

Using fecal microbiota analysis, a prospective cross-sectional exploratory study in ER+/HER2- metastatic breast cancer patients identified gut microbial taxa associated with response to CDK4/6 inhibitors. Studies indicate that the abundance of *Clostridium innocuum*, *Oscillibacter ruminantium*, and *Eubacterium hallii* positively correlates with the neutrophil-to-lymphocyte ratio (NLR), suggesting these microbes may negatively impact CDK4/6 inhibitor efficacy. Notably, higher levels of *Roseburia faecis* were associated with more favorable prognosis, indicating its potential role in enhancing treatment. These findings point to both microbial biomarkers for CDK4/6 inhibitor response and the potential for gut microbiota modulation to improve outcomes [143].

Microbes and their metabolites show great potential for the development of novel therapeutics, including small-molecule drugs, targeted therapies, and immunotherapies [149]. Sodium butyrate, either alone [150] or in combination with other anticancer drugs such as trastuzumab [151] and crizotinib [152], has demonstrated promising anti-tumor activity in breast cancer patients. A recent study found that commensal *Lactobacillus johnsonii* collaborates with *Clostridium* to promote the production of indole-3-propionic acid and activate precursor exhausted T cells (Tpex) in pan-cancer (including BC), thereby enhancing the effectiveness of immune checkpoint blockade (ICB) therapy [153].

Radiotherapy can affect the abundance of tumor microbiota, and the microbiota can also influence tumor response or resistance to radiotherapy by affecting TME [145]. Commensal fungi promote the differentiation of Th2 cells mediated by Dectin-1, thereby creating an immunosuppressive TME that may facilitate cancer progression and the development of radiotherapy resistance. The use of antibiotics to deplete fungi can lead to an improvement in the efficacy of radiotherapy. In contrast, commensal bacteria exert opposing effects: they can inhibit fungal proliferation and promote post-radiotherapy T cell activation, thereby enhancing treatment efficacy [144]. The gut microbiota and its metabolites play a role in radiotherapy, with evidence showing that acetate, butyrate, indole-3-carboxaldehyde, and kynurenic acid can enhance the response to radiotherapy and reduce its side effects [154]. Nevertheless, the intricate interplay between the microbiota and radiotherapy is not fully elucidated and warrants further investigation.

### 5.2. Targeting Microorganisms to Treat or Reduce Complications

Modulating the gut microbiota composition represents one of the most cost-effective strategies for harnessing the microbiome in breast cancer treatment [155] (Table 5). Key approaches include dietary control and the use of prebiotics or probiotics. Additionally, fecal microbiota transplantation (FMT) and judicious use of antibiotics can also help regulate microbiota composition.

Dietary intervention is a foundational microbiome-modulating approach due to its rapid, reversible impact on intestinal and breast microbiota [168]. Changes in dietary structure can lead to alterations in microbes such as *Acidaminococcus*, *Tyzzerella*, and *Hungatella*, which are associated with breast cancer risk [169]. Compared to Western dietary patterns, adherence to the Mediterranean diet can reduce the incidence of all subtypes of breast cancer and decrease the risk of breast cancer recurrence [170,171]. Studies have found a significant increase in the abundance of *Lactobacilli* and *Bifidobacterium* content in the breast among populations following the Mediterranean diet [172], demonstrating the diet’s ability to modulate the breast microbiota community.

Prebiotics and probiotics are employed to promote the colonization of beneficial microbiota and to influence the concentration of active drug metabolites. Prebiotics, typically composed of indigestible food components like fiber, are used to specifically stimulate the growth or activity of intestinal microbial populations. For example, inulin is used to stimulate the growth of *Bifidobacterium* in the gut [173]. Common probiotics such as *Lactobacilli* and *Bifidobacterium* help reduce local and systemic inflammation, enhance the intestinal epithelial barrier, and competitively exclude pathogens. *Lactobacillus acidophilus* (*L. acidophilus*), a probiotic strain, modulates the gut microbiota in tumor-bearing mice and elicits a protective Th1 immune response, thereby enhancing anti-tumor immunity [134]. Suppression of tumor cell dissemination and lung metastasis has also been observed, potentially linked to immunomodulatory mechanisms via reduced macrophage infiltration in both tumors and lungs [174]. Additionally, probiotics have been shown to produce antimicrobial substances, thereby promoting anti-inflammatory responses in gut-associated lymphoid tissue.

Transferring fecal microbiota from a healthy donor to the patient’s intestines, known as FMT, is used to restore microbial balance. Compared to other treatment methods, FMT has significant advantages because it can maintain microbial diversity without disrupting natural gut ecology. Clinically, *Clostridium difficile* infection is treated with FMT [161]. Beyond this, it holds promise for reversing gut microbiota caused by antibiotics, and chemotherapy is expected to be reversed by FMT [160]. However, there is no definitive evidence linking FMT to BC therapy directly.

The use of antibiotics in cancer prevention and treatment remains controversial. Their use can significantly affect the effectiveness of BC treatment. A clinical study found that in 120 patients receiving neoadjuvant therapy for HER2-positive breast cancer with anthracyclines, taxanes, and CTX (as well as trastuzumab), the use of antibiotics after the start of treatment was associated with a poorer response rate and disease-free survival, possibly by affecting the dysbiosis of the intestinal microbiota and thereby influencing the effectiveness of neoadjuvant therapy [175]. Animal studies suggest that vancomycin and streptomycin may alter the composition of gut microbiota (e.g., key phyla such as *Actinobacteria*, *Bacillota*, *Bacteroidota*, and *Clostridia*) and their metabolites, thereby modulating the local TME. These changes ultimately lead to significantly reduced efficacy of trastuzumab in HER2-positive breast cancer mouse models [176]. Conversely, appropriate use of antibiotics can eliminate pathogenic bacteria and control inflammation. In mouse models, aerosolized antibiotics or periodic oral administration of benzylpenicillin can regulate local microbial communities, eradicate *S. epidermidis*, enhance tumor immunity, reduce the invasiveness of breast cancer, and enhance the chemotherapeutic efficacy of paclitaxel [81]. Achieving a balance between selectively eliminating harmful microbes and preserving beneficial ones remains a significant challenge.

Beyond compositional modulation, engineered microbes enable precise therapeutic targeting. For instance, some microbes, such as attenuated *Salmonella*, have been employed for non-toxic colonization of tumors, combined with the delivery of cytokines (IL-15) and immune checkpoint inhibitors (anti-CTLA-4 and anti-PD-L1), reducing tumor growth and improving survival in mouse models [51]. Engineered probiotics can selectively guide chimeric antigen receptor (CAR)-T cells to tumor sites and enhance immune cytotoxicity [163]. *Listeria monocytogenes* has shown potential for in vivo gene delivery for cancer treatment [156]. In mouse models, administration of attenuated *Mycobacterium obuense* can activate tumors, downregulate the expression of cancer genes such as CLDN3, shrink tumor volume, and reduce the invasiveness of breast cancer [162]. Oncolytic bacteria and viruses are also a novel class to treat breast cancer [157,164]. Their toxicity profiles are limited and typically non-overlapping with the toxicity associated with other treatment methods. Multiple oncolytic viruses have entered clinical trials, and they have demonstrated positive effects both when used as monotherapy (HF10 [177], vvDD-CDSR [178], etc.) and combination therapy (T-VEC/Talimogene Laherparepvec [179], etc.). Multiple bacteriophage systems demonstrate significant potential in vaccine development, targeted drug delivery, and disease diagnosis for breast cancer. Moreover, their ability to directly infect and eliminate pathogenic bacteria also provides unique value for bacteriophage-based therapeutic strategies [180]. For example, Shoae-Hassani et al. developed apoptin-expressing λ phage nanoparticles that target her-2-overexpressing breast cancer cells and significantly inhibit their proliferation in vitro/vivo [181]. Bacterial vaccines offer a novel approach for precise and effective intervention against specific bacteria within tumors. These vaccines can stimulate the immune system to produce antibodies and activate immune cells capable of recognizing and combating targeted bacteria or closely related bacterial groups. Leveraging the characteristic of *F. nucleatum* to colonize BC cells, researchers have developed a bacterial-derived outer membrane vesicle (OMV)-coated nanoplatform that precisely targets tumor tissues, enabling dual eradication of *F. nucleatum* and cancer cells. This strategy effectively transforms intratumoral bacteria into immune enhancers for TNBC immunotherapy [165].

Furthermore, the composition of microbiota is also associated with the toxicity and side effects of chemotherapy drugs [182]. Studies have revealed that reduced α-diversity of the gut microbiota and decreased abundance of specific microbes (e.g., *Faecalibacterium*) prior to chemotherapy are associated with chemotherapy-induced gastrointestinal adverse effects [166]. In a systematic review and meta-analysis, probiotic combinations of *Lactobacillus*, *Bifidobacterium*, and *Streptococcus* plus fructooligosaccharides reduce pro-inflammatory TNF-α levels, alleviate edema volume and improve quality of life among patients with breast cancer-related lymphedema [158]. Additionally, the use of *Lactobacillus* can reduce bone marrow suppression and immune suppression induced by chemotherapy in mice [167]. Archaeosomes can serve as drug carriers to transport paclitaxel for better therapeutic effects and reduced side effects [183]. Thus, utilizing probiotics to modulate intestinal microbiota or inhibit related enzyme activity holds significant promise for alleviating chemotherapy-related adverse effects and improving patient prognosis [184,185].

## 6. Conclusions and Perspective

The human microbiome exerts profound and complex influences on both physiological and pathological processes. It participates not only in key physiological functions such as digestion, development, and immunity but can also provoke local or systemic inflammation and even drive tumorigenesis. In the prevention and treatment of breast cancer, understanding the various components of the immune microenvironment and their regulatory balance is essential.

Evidence suggests that the breast microbiome contributes to shaping the tumor microenvironment through multiple pathways, including inducing DNA damage, interfering with estrogen metabolism, triggering chronic inflammation and immunosuppression, and producing carcinogenic metabolites. However, research in this field remains relatively limited due to the low biomass of microorganisms in breast tissue and susceptibility to contamination. The gut microbiome has also gained attention as a distal regulatory hub linking inflammation to breast cancer. Although anatomically distant from the breast, elucidating the signaling pathways that connect gut microbial activity to breast carcinogenesis is of considerable importance. While the regulatory effects of the gut microbiome on the immune system have been extensively studied, its specific role in breast cancer progression requires further investigation.

Advances in microbiomics and metagenomics are enabling more precise identification of microbial communities under specific conditions. A key unresolved question is whether the microbiome initiates breast cancer through inflammation or whether the tumor microenvironment creates a pro-inflammatory niche that selects for certain microbial populations. Larger-scale studies, combined with multi-omics technologies, will be necessary to clarify these complex interactions and underlying mechanisms.

In-depth analysis of microbiome–tumor microenvironment interactions hold promise for identifying novel biomarkers for breast cancer prevention, diagnosis, and treatment, as well as revealing new therapeutic targets. However, translating microbiome-based strategies into clinical practice faces significant challenges, including developing methods to precisely target tumor-associated microbiota and understanding the potential physiological consequences of intentional microbial manipulation.

## Figures and Tables

**Table 1:** Differential Bacterial Taxa by Breast Tissue Subtype and Normal Tissue (at Family/Genus/Species Levels).

Tissue Type	Adjacent vs. Healthy Control Tissue	Tumor vs. Healthy Control Tissue	Tumor vs. Adjacent Tissue
ER+	Increased: *Arcanobacterium*, *Bifidobacterium*, *Cardiobacterium*, *Citrobacter*, *Escherichia* [24] Decreased: *Vibrio*, *Pseudoalteromonas*, *Photobacterium*, *Marinobacterium*, *Prevotella*_9 [25]	Decreased: *Alkanindiges*, *Micrococcus*, *Caulobacter*, *Proteus*, *Brevibacillus*, *Kocuria*, *Parasediminibacterium* [20] (more abundant in non-tumor tissues)	
PR+		Increased: *Pelomonas*, *Ralstonia*, *Oblitimonas*, *Lactobacillus*, *Methylophilus*, *Achromobacter* [20] (within tumor tissue)	
TNBC	Increased: *Aerococcus*, *Arcobacter*, *Geobacillus*, *Orientia*, *Rothia* [24]	Increased: *Azomonas*, *Alkanindiges*, *Caulobacter*, *Proteus*, *Brevibacillus*, *Kocuria*, *Parasediminibacterium* [20] (within tumor tissue) Decreased: Cutibacterium, Pseudoalteromonas, Photobacterium [25]	Increased: Sphingomonas, Sphingomonadaceae family [26] Decreased: *Sporosarcina* [26]
HER2+	Increased: *Acinetobacter*, *Pseudomonas*, *Stenotrophomonas*, *Cutibacterium* [25]	Increased: *Cloacibacterium*, PRD01a011B, *Alloprevotella*, *Stakelama*, *Filibacter*, *Blastomonas*, *Anaerostipes* [20] (within tumor tissue) Decreased: *Glaciecola*, *Vibrio*, *Photobacterium*	Increased: Sphingomonas [26]
TPBC	Increased: *Bordetella*, *Campylobacter*, *Chlamydia*, *Chlamydophila*, *Legionella*, *Pasteurella* [24]		
Luminal A			Increased: *Corynebacterium* [26]
Luminal B			Increased: *Alloiococcus* [26]
BC	Increased: *Staphylococcus*, *Acinetobacter*, *Burkholderia–Caballeronia–Paraburkholderia*, *Escherichia–Shigella*, *Shewanella*, *Mycoplasma*, Clostridium_sensu_stricto_7, *Psychrobacter*, *Wolbachia*, *Glaciecola* [25]	Increased: *Pseudomonadaceae* and Enterobacteriaceae, *Porphyromonas* and *Azomonas* [20] (significantly higher in tumor tissue); *Actinomyces*, *Bartonella*, *Brevundimonas*, *Coxiella*, *Mobiluncus*, *Mycobacterium*, *Rickettsia*, *Sphingomonas* [24]	Increased: Peptostreptococcales_Tissierella family and *Finegoldia*, *Rothia genus*; *Streptococcus*, *Rothia*, and *Staphylococcus* [26] Decreased: Pseudomonadaceae family and *Pseudomonas* genus; Enterobacteriaceae family [26]
Tumor Adjacent Normal Tissue	Increased: *Bacillus*, *Staphylococcus*, Enterobacteriaceae (unclassified), Comamondaceae (unclassified), and Bacteroidetes (unclassified) [18]		
Healthy Control Tissue	Healthy Tissue Signature: *Propionibacterium*, *Staphylococcus* (abundant in non-tumor tissues) *Finegoldia*, *Granulicatella*, *Streptococcus*, *Anaerococcus*, Ruminococcaceae UCG-002, *Corynebacterium 1*, *Alicyclobacillus*, *Odoribacter*, *Lactococcus*, *Esherichia/Shigella* [20]	Increased: *Rickettsia*, *Acinetobacter*, *Ralstonia*, *Delftia*, *Arthrobacter*, *Stenotrophomonas* [25]; *Prevotella*, *Lactococcus*, *Streptococcus*, *Corynebacterium*, *Micrococcus* [18]	

**Table 2:** Key PRRs Implicated in Breast Cancer Microbiome Sensing.

PRR Family	Member(s)	Ligand/Microbial Component	Relevance in Breast Cancer
TLRs	TLR1, TLR2, TLR6	Lipoproteins (Gram-positive bacteria)	Differentially expressed across subtypes; associated with tumor progression
	TLR4	LPS (Gram-negative bacteria)	High expression correlates with poor prognosis; modulates immune microenvironment
	TLR5	Flagellin (bacterial motility protein)	Involved in bacterial sensing; potential link to microbiome-driven inflammation
	TLR3	Double-stranded RNA (viral/bacterial)	Expression negatively correlates with patient survival
	TLR9	Unmethylated CpG DNA (bacterial/viral)	Elevated in some BC tissues; prognostic implications
NLRs	NOD1	Peptidoglycan fragments	Participate in intracellular bacterial sensing; may influence BC cell signaling
RLRs	RIG-I, MDA5	Viral RNA	Primarily antiviral; potential role in viral-associated BC hypotheses
FPR	FPR1	Bacterial formylated peptides	Loss-of-function variant (Rs867228) linked to infection risk and luminal B BC susceptibility

**Table 3:** Impacts of Microbial Metabolites on Breast Cancer.

Metabolite		Source	Effects on Breast Cancer	Mechanism	Ref.
LCA		Aerobic floras (Mostly *Clostridia*)	Influence macrophage polarization and T cell function	Act via MAPK pathway and TGR5 receptors; decreased in early-stage breast cancer	[107]
SCFAs	Acetate	*Blautia*	Augment anti-tumor immunity and inhibit metastasis.	Enhance CD8+ T cell infiltration	[111]
	Propionate	Gut microbiota	Inhibit JAK2/STAT3, induce apoptosis by promoting ROS levels and MAPK pathway activation	Causes cell-cycle arrest, increases ROS, phosphorylates p38 MAPK	[112]
	Butyrate	Gut microbiota	Regulate immune response, lower inflammation, enhance anti-PD-1 therapy effectiveness	Binds to GPR109A, influences T cell differentiation	[113,114]
Lactate		*Lactobacilli*	Influence macrophage polarization and T cell function.	Modulate immune cell function; dysregulation leads to immunosuppressive TME.	[37,115]
Folate		Gut microbiota	Enhance bone metastasis risk	Impact DNA methylation, repair, and synthesis	[116]
LPS		Gram-negative bacterial cells (breast cancer)	Suppress tumor immunity to enable tumor growth and metastasis	Downregulation of Akt/GSK3β/β-Catenin signaling pathway; recruit MDSCs via LPS/S100A7/TLR4 and impair TLR4-mediated tumor immunity	[117,118,119]
EVs		*F. nucleatum* (breast microbiota)	promote BC cell proliferation	EVs/TLR4	[76]
		Probiotics	Exert anti-inflammatory properties	/	[120]

**Table 4:** Microbial Impacts on Breast Cancer Therapeutic Efficacy.

Therapy Category		Microbial Element	Specific Mechanism	Measurable Outcome	Ref.
Chemotherapy	Gemcitabine	Mycoplasma	Upregulates cytidine deaminase	Reduction in drug activation	[138]
	CTX	Specific gut bacteria	Enhances the anti-tumor immune response by inducing Th17 and Th1 cells	Increase in tumor-infiltrating T cells	[139,140]
	Platinum agents	Specific gut bacteria	Generates ROS	DNA damage and cell apoptosis	[141]
	Docetaxel	*A. muciniphila*	-	-	[142]
Immunotherapy	CDK4/6 inhibitor	*Clostridium innocuum*, *Oscillibacter ruminantium*, and *Eubacterium hallii*	Negatively correlated	Neutrophil-to-lymphocyte ratio	[143]
		*Roseburia faecis*	Positively correlated		
	Anti-PD-1 therapy	Gut microbiota	Production of the metabolite butyrate	-	[113]
Radiotherapy		Opposing effects of commensal fungi and bacteria	Activates Dectin-1 pathway	Promotion of Th2 cell differentiation	[144,145]

**Table 5:** Microbiome-Targeting Strategies and Their Applications in Breast Cancer.

Strategy		Advantages	Limitations	Applications	Ref.
Dietary Modifications		Non-invasive, low-cost, reversible	Slow effects, inter-individual variability	Mediterranean diet	[156,157]
Probiotics/Prebiotics		Targets beneficial strains	Strain-specific, colonization challenges	Inulin; *Lactobacilli* and *Bifidobacterium*	[158,159]
FMT		Rapid restoration of microbial diversity, safety (infection, immune reactions)	Not supported by clear evidence	Post-antibiotic/chemotherapy recovery	[160,161]
Antibiotics		Rapid pathogen clearance	Disrupts commensal microbiota, may reduce efficacy	Selective use for efficacy enhancement or infection control	[81]
Engineered Microbes	Genetically Modified Bacteria	Metabolic adjustment, multifunctional delivery	Technical complexity, regulatory hurdles, safety risks	Targetedtherapy, precisiondrug/genedelivery, multifunctional	[156,162,163]
	Oncolytic bacteria and viruses	Complementary mechanisms of action, high target specificity			[157,164]
	Bacterial vaccines	Dual targeting (bacteria and tumor)			[165]
Treatment-related adverse effects	Gastrointestinal Toxicity		Regulation of the intestinal microenvironment	gutmicrobiota (e.g., Faecalibacterium)	[166]
	Lymphedema			ProbioticCombination (*Lactobacillus*, *Bifidobacterium*, *Streptococcus* + Fructooligosaccharides)	[158]
	Myelosuppression and immunosuppression			*Lactobacillus*	[167]

## Data Availability

No new data were created or analyzed in this study. Data sharing is not applicable to this article.

## Abbreviations

The following abbreviations are used in this manuscript:

*A. muciniphila*	*Akkermansia muciniphila*
AP-1	activator protein 1
BC	breast cancer
*B. burgdorfer*	*Borrelia burgdorferi*
BFT	*B. fragilis* toxin
CTX	cyclophosphamide
DCs	dendritic cells
EBV	Epstein–Barr virus
*E. coli*	*Escherichia coli*
EMT	epithelial–mesenchymal transition
ETBF	enterotoxigenic Bacteroides fragilis
FMT	fecal microbiota transplantation
*F. nucleatum*	*Fusobacterium nucleatum*
FPR1	formyl peptide receptor-1
HCMV	human cytomegalovirus
*H. hepaticus*	*Helicobacter hepaticus*
HMTV	human mammary tumor virus
HPV	human papillomavirus
ICB	immune checkpoint blockade
IHC	immunohistochemical
*L. acidophilus*	*Lactobacillus acidophilus*
LCA	lithocholic acid
LPS	lipopolysaccharides
MALT	mucosa-associated lymphoid tissue
MDSCs	myeloid-derived suppressor cells
*M. globosa*	*Malassezia globosa*
NLRs	NOD-like receptors
OS	overall survival
OXA	oxaliplatin
PAMPs	pathogen-associated molecular patterns
PD-L1	programmed cell death ligand 1
*P. gingivalis*	*Porphyromonas gingivalis*
PRRs	pattern recognition receptors
RNS	reactive nitrogen species
ROS	reactive oxygen species
SCFAs	short-chain fatty acids
*S. epidermidis*	*Staphylococcus epidermidis*
TLRs	Toll-like receptors
TME	tumor microenvironment
TNBC	triple-negative breast cancer
